# Effects of cytosine modifications on DNA flexibility and nucleosome mechanical stability

**DOI:** 10.1038/ncomms10813

**Published:** 2016-02-24

**Authors:** Thuy T. M. Ngo, Jejoong Yoo, Qing Dai, Qiucen Zhang, Chuan He, Aleksei Aksimentiev, Taekjip Ha

**Affiliations:** 1 Center for Biophysics and Quantitative Biology, University of Illinois at Urbana-Champaign, Urbana, Illinois 61801, USA; 2 Department of Physics and Center for the Physics of Living Cells, University of Illinois at Urbana-Champaign, Urbana, Illinois 61801, USA; 3 Department of Chemistry, The University of Chicago, Chicago, Illinois 60637, USA; 4 Department of Biochemistry and Molecular Biology, The University of Chicago, Chicago, Illinois 60637, USA; 5 Institute for Biophysical Dynamic, The University of Chicago, Chicago, Illinois 60637, USA; 6 Howard Hughes Medical Institute, Chicago, Illinois 60637, USA; 7 Howard Hughes Medical Institute, Baltimore, Maryland 21205, USA; 8 Department of Biophysics and Biophysical Chemistry, Johns Hopkins University, Baltimore, Maryland 21205, USA; 9 Department of Biophysics, Johns Hopkins University, Baltimore, Maryland 21205, USA; 10 Department of Biomedical Engineering, Johns Hopkins University, Baltimore, Maryland 21205, USA

## Abstract

Cytosine can undergo modifications, forming 5-methylcytosine (5-mC) and its oxidized products 5-hydroxymethylcytosine (5-hmC), 5-formylcytosine (5-fC) and 5-carboxylcytosine (5-caC). Despite their importance as epigenetic markers and as central players in cellular processes, it is not well understood how these modifications influence physical properties of DNA and chromatin. Here we report a comprehensive survey of the effect of cytosine modifications on DNA flexibility. We find that even a single copy of 5-fC increases DNA flexibility markedly. 5-mC reduces and 5-hmC enhances flexibility, and 5-caC does not have a measurable effect. Molecular dynamics simulations show that these modifications promote or dampen structural fluctuations, likely through competing effects of base polarity and steric hindrance, without changing the average structure. The increase in DNA flexibility increases the mechanical stability of the nucleosome and vice versa, suggesting a gene regulation mechanism where cytosine modifications change the accessibility of nucleosomal DNA through their effects on DNA flexibility.

## Abstract (web summary)

Cytosine modifications are important epigenetic markers yet their physical influence on DNA is not well understood. Here, Ngo *et al*. show that different alterations affect DNA flexibility, suggesting a mechanism where modifications change accessibility of nucleosome bound DNA.

## Graphical abstract

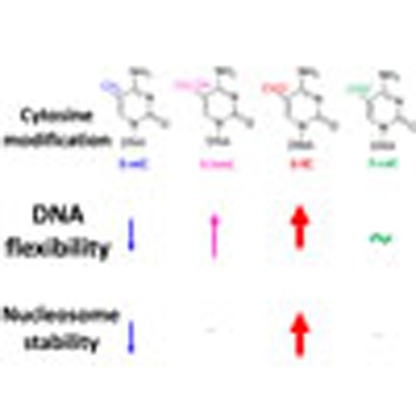

Canonical DNA comprises four nucleobases: adenine (A), thymine (T), cytosine (C) and guanine (G). It has long been recognized that cytosine can undergo chemical modifications at the fifth carbon of its pyrimidine ring (5-C)^1^,^2^,^3^. In mammals, new DNA methylation (5-methylcytosine; 5-mC) is established by transferring the methyl group from *S*-adenosylmethionine to cytosine at a CpG site by DNA methyltransferases DNMT3A and DNMT3B (ref. 1). DNA methylation is stable in somatic cells. However, DNA methylation is erased in specific developmental stages such as preimplantation embryos and developing primordial germ cells^4^,^5^. The loss of DNA methylation is required for setting up pluripotent states in embryos and for erasing parental methylation imprints in developing germ cells^6^. In addition to passive dilution due to DNA replication, cytosine methylation marks can be erased through an active demethylation pathway involving three sequential steps of oxidation of 5-mC to 5-hydroxymethylcytosine (5-hmC), to 5-formylcytosine (5-fC) and then to 5-carboxylcytosine (5-caC) performed by TETs enzymes, followed by base excision of 5-fC or 5-caC by DNA glycosylase and base excision repair to convert an abasic site to cytosine^6^,^7^,^8^,^9^,^10^,^11^,^12^,^13^.

5-mC is found in most plants, animals and fungi, and has a profound effect on genome stability, gene expression and development^1^,^14^. 5-hmC, 5-fC and 5-caC may also function in gene regulation^6^. For instance, 5-hmC is preferentially enriched at distal regulatory elements such as enhancers in mouse or human embryonic stem cells^15^,^16^,^17^,^18^, whereas 5-fC or 5-caC tend to accumulate at poised enhancers and promoters^19^,^20^. Possible mechanisms for the regulatory role of cytosine modifications include the following: (1) altering the physical properties of DNA; (2) modulation of chromatin accessibility; and (3) recruitment of proteins that recognize cytosine modifications as their substrates followed by transcriptional activators/repressors that translate the presence of modifications to downstream signalling of transcription machinery^14^,^21^.

DNA flexibility was shown to affect the binding of proteins to methylated DNA and DNA-containing lesion^22^,^23^,^24^,^25^. For instance, a base-pair mismatch increases DNA flexibility, facilitating the recognition by repair proteins such as DNA glycosylases and MutS (ref. 25). CpG methylation reduces DNA flexibility affecting the binding of some proteins to their cognitive sequences such as the EcoRI restriction site or the cAMP DNA responsive element^22^,^23^. 5-hmC was shown to destabilize duplex DNA structure^26^. However, little is known about the effects of 5-hmC, 5-fC and 5-caC modifications on DNA flexibility.

In eukaryotes, DNA is packaged into a basic unit, a nucleosome, which consists of 147 bp of DNA wrapped around a histone octamer core^27^. Stable packing of DNA in nucleosomes imposes a barrier against replication, transcription and repair^28^,^29^,^30^. The nucleosomal DNA can be made accessible by partial unwrapping of DNA from the histone core^31^,^32^, which can occur spontaneously or induced by tension. Highly dynamic chromatin anchored to various subnuclear structures is likely to experience tension. Cytosine modifications may affect gene regulation by changing nucleosome stability and unwrapping. For example, 5-mC has been reported to affect the nucleosome structure^33^,^34^,^35^. However, how cytosine modifications including 5-mC and its oxidation products (5-hmC, 5-fC and 5-caC) affect nucleosome unwrapping under tension is not known.

Previously, we suggested that DNA sequence may affect gene expression and other processes through their effects on DNA flexibility and resulting alternations in nucleosome unwrapping^36^. We found that the more flexible the DNA sequence is, the more stably the DNA binds to the histone core under a physiologically relevant level of tension applied to the DNA ends. Whether the relationship connecting higher flexibility of DNA to higher mechanical stability of the nucleosome holds also for DNA modifications is yet to be tested.

Here we report a comprehensive survey of the effect of cytosine modifications on DNA flexibility using a single-molecule cyclization assay: 5-fC remarkably increases DNA flexibility while 5-mC reduces and 5-hmC enhances flexibility, and 5-caC does not have a measurable effect. Molecular dynamics simulations elucidate the microscopic mechanism of the DNA flexibility changes induced by cytosine modifications. Finally, we examine the correlation between nucleosome mechanical stability and DNA flexibility modulated by cytosine modifications using a combination of optical tweezers and single-molecule fluorescence resonance energy transfer (smFRET). 5-fC increases while 5-mC reduces nucleosome mechanical stability, corresponding to the increase and decrease in DNA flexibility induced by 5-fC and 5-mC, respectively.

## Results

### Effect of cytosine modifications on DNA flexibility

**Figure 1:**
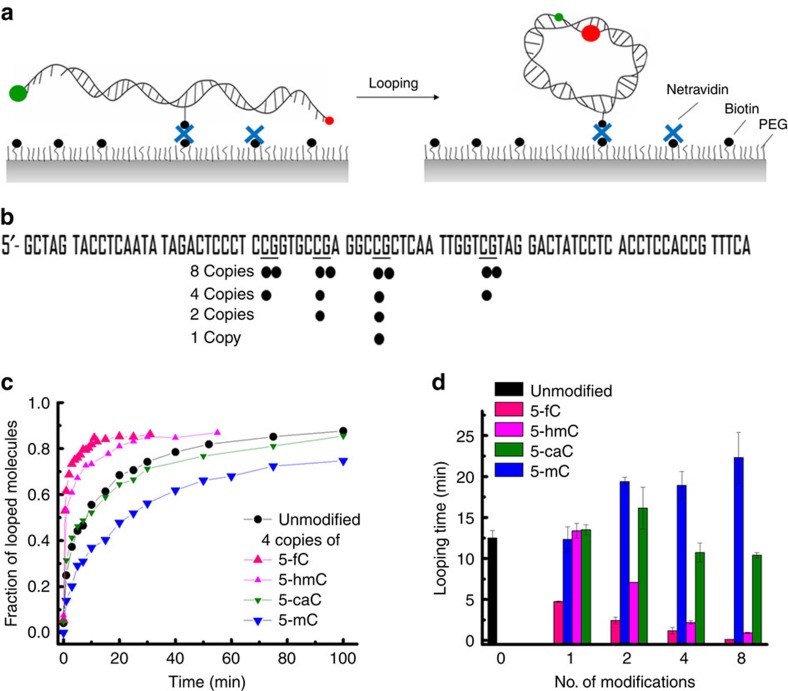
Effect of cytosine modifications on DNA flexibility. (**a**) Schematic representation of single-molecule DNA cyclization assay. DNA fragments are immobilized on a microscope slide. A FRET pair is incorporated at the two sticky ends of DNA. High-FRET population is monitored over time to quantify the fraction of looped DNA. If DNA is more flexible, it takes less time for loop formation. (**b**) DNA sequence used. Modified CpG sites are underscored. The locations of cytosine modifications in the constructs containing multiple modifications are indicated as black dots. (**c**) Fraction of looped molecule as a function of time for unmodified DNA and DNA containing four copies of 5-fC, 5-hmC, 5-caC and 5-mC. (**d**) Looping time versus the number of modifications per construct. Error bars represent the s.e.m. (*n*≥3).

The DNA cyclization assay was introduced by Shore *et al*.^37^ as a measure of the DNA's mechanical properties. Here we used a single-molecule DNA cyclization assay^38^ to quantify the effect of cytosine modification on DNA flexibility. In this assay (Fig. 1a), DNA molecules terminated with two complementary 5′ overhangs of 10 nucleotides each were immobilized on a polyethylene glycol (PEG)-coated quartz slide using the biotin–neutravidin interaction. A fluorescence resonance energy transfer (FRET) pair at the 5′-ends enabled the detection of loop formation (or cyclization) via an increase in FRET. The circumference of the closed DNA loop was 90 bp; cytosine modifications were introduced near the middle of the construct to maximize the contrast. After rapidly increasing the concentration of NaCl from 10 mM to 1 M, the fraction of high-FRET population corresponding to the looped DNA was determined as a function of time. Here we are using the rate of loop formation as an operational measure of DNA flexibility^36^. If DNA is more flexible, it forms a loop faster and vice versa. Loop formation is reversible, but in all constructs tested, the equilibrium population of the looped state was above 70%. Therefore, as a measure of the looping time, we used the apparent looping time obtained by fitting the looped fraction versus time with an exponential function.

DNA oligonucleotides containing various cytosine modifications at prescribed locations were synthesized (Fig. 1b). Two strands of the DNA construct for single-molecule cyclization were ligated from the modified biotin/fluorophore-labelled oligos and purified separately to ensure complete ligation of the two strands.

Figure 1c shows the looped fraction versus time for constructs containing four copies of modified cytosines and a construct without cytosine modification. The 5-mC modifications resulted in slower looping, 5-fC and 5-hmC modifications accelerated looping, whereas 5-caC did not have a detectable effect. The magnitudes of the effect varied greatly among different modifications. For a single modification, 5-mC or 5-hmC had almost no effect on the looping time, whereas 5-fC reduced the looping time by threefold (Fig. 1d). With two or more copies of cytosine modification, 5-hmC significantly decreased the looping time, whereas 5-mC measurably increased it. Thus, multiple copies of the same modifications magnify the effect. Similar reduction in DNA flexibility was observed when DNA was methylated using methyltransferase M.SssI (Supplementary Fig. 1). Please note that our looping assay is more sensitive to local increases of DNA flexibility than to local decreases. Overall, our data show that the 5-mC modification decreases DNA flexibility, whereas 5-fC or 5-hmC increases DNA flexibility, and the magnitude of the effects is the largest for 5-fC and smaller for 5-hmC or 5-mC.

### Structural fluctuations result in DNA flexibility change

**Figure 2:**
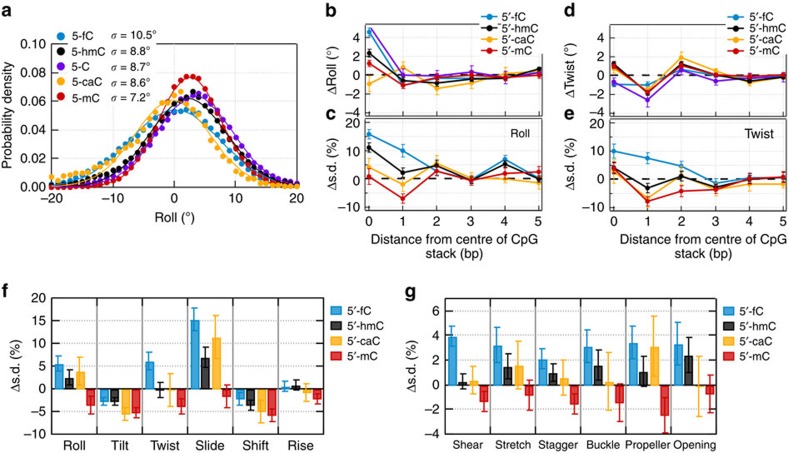
Molecular dynamics simulations of DNA with cytosine modifications. (**a**) Simulated distributions of the roll parameter in a CpG step for four cytosine variants (blue symbols, 5-fC; black, 5-hmC; orange, 5-C; red, 5-mC). A solid line shows a Gaussian fit to a data set. The s.d.'s are provided in the legend. (**b**,**c**) The change in the mean value of roll (**b**) and its s.d. (**c**) versus the distance from the centre of the CpG step. For each cytosine modification, the change was computed relative to the values observed for unmodified DNA. Each data point represents an average over the four CpG steps and the corresponding molecular dynamics trajectory block-averaged with a 20-ns interval. The error bars are the s.e.m. (**d**,**e**) Same as in **b** and **c** but for the twist parameter. (**f**,**g**) The average change in the s.d. of the inter-base-pair (**f**) and intra-base-pair (**g**) structural parameters relative to the corresponding values of unmodified DNA. Each data point represents an average over 3 bp centred at a CpG step, over the four CpG steps and over the corresponding molecular dynamics trajectory block-averaged with a 20-ns interval. The error bars are the s.e.m.

To determine the mechanism of DNA flexibility modulation by cytosine modifications, we carried out molecular dynamics simulations of the central 70-bp fragment of the experimental DNA construct in 1 M NaCl solution at room temperature (Fig. 1b). In total, five systems were built: four systems each featuring eight copies of 5-mC, 5-hmC, 5-caC or 5-fC modifications, and one system without any modifications. Each system was simulated for ∼250 ns and the instantaneous coordinates of each system were recorded every 4.8 ps. The ensembles of the atomic coordinates were analysed using the 3DNA programme^39^, to characterize the DNA conformations in terms of the inter-base-pair (roll, tilt, twist, slide, shift and rise) and intra-base-pair (shear, stretch, stagger, buckle, propeller and opening) structural parameters. For all base pairs in all DNA constructs, the distributions of the instantaneous structural parameters were Gaussian. Cytosine modifications shifted the peak position and width of the distribution (Fig. 2a). In general, broadening of a distribution would indicate an enhanced flexibility of the molecule, however, precise mechanistic interpretation depends on the type of the structural parameter.

Out of the 12 parameters, roll (an inclination angle between two stacked base-pair planes) and twist (a relative torsion of two stacked base pairs with respect to the helical axis) are closely related to bending^40^. Thus, one explanation for the modulation of the looping probability on cytosine chemical modification is a change in the intrinsic curvature of the DNA molecule, which would manifest itself as a change of the twist and roll parameters^40^. In our simulations, the 5-fC, 5-hmC, 5-caC and 5-mC modifications increased the roll by 5°, 2°, −1° and 1° at CpG steps, respectively (Fig. 2b). The effects of cytosine modifications on the twist (Fig. 2d) and on other parameters (Supplementary Fig. 2) were even less significant or negligible, suggesting that the chemical modifications do not induce significant static structural deformation.

Another explanation is that cytosine modifications change the looping kinetics by increasing or decreasing the amplitudes of fluctuations around the average structure^26^. Bulky nonpolar methyl groups can suppress local structural fluctuations of DNA by limiting the stochastic motion of the polar bases^22^. Conversely, bulky but polar hydroxymethyl groups can enhance local fluctuations if the increased polarity of the modified bases overcompensates for their larger steric footprint^26^. Formyl group, which is much more electron withdrawing compared with the hydroxymethyl group, can be expected to enhance the local flexibility of DNA even more due to reduced base-pairing ability. Compared with the formyl groups, the additional negative charges of the carboxylate groups can be expected to increase the internal electrostatic repulsions within DNA and thereby reduce its flexibility.

The results of our molecular dynamics simulations support this conjecture. For example, the s.d.'s of both roll and twist increase on introduction of 5-fC at the CpG sites (Fig. 2c,e). The effects of 5-hmC and 5-caC are less significant than that of 5-fC, and the effect of 5-mC is opposite (Fig. 2c,e)—the fluctuations are damped. Cytosine modifications affected the structural fluctuations of several (up to six) neighbouring base pairs (Fig. 2c,e), suggesting that chemical modifications of multiple CpG sites in close proximity may alter the local flexibility of DNA in a cooperative manner. Except tilt and shift, the s.d.'s of the other eight structural parameters were found to rank in the same order (5-fC>5-hmC≳5-caC≳unmodified C>5-mC) as for roll and twist with some mixed ordering among 5-hmC, 5-caC and unmodified C (Fig. 2f,g and Supplementary Figs 2 and 3). For tilt and shift, a slightly different order was observed (unmodified C>all others), indicating the dominance of steric restrictions over polarity in determining the variation of these two parameters. A test simulation of the DNA fragment containing protonated (uncharged) 5-carboxylcytosine modifications showed significant increases in the s.d.'s of all structural parameters relative to the 5-caC values (Supplementary Fig. 4). Thus, the modest effect of the 5-caC modifications on the DNA flexibility results from the cancellation of effects associated with the increased polarity and the negative charge of the modified base. Overall, molecular dynamics simulations show that local structural fluctuations are significantly increased by 5-fC, marginally increased or unaffected by 5-hmC and 5-caC, and reduced by 5-mC without significantly changing the average DNA structure, correlating well with our experimental measurements of DNA flexibility.

### 5-mC loosens packing of nucleosomal DNA ends

Biophysical studies have shown that 5-mC can change nucleosome structure and dynamics^24^,^33^,^34^,^35^. Using smFRET, Choy *et al*.^34^ provided evidence that DNA methylation induces tighter wrapping of DNA around the histone core. In contrast, using ensemble FRET and small-angle X-ray scattering, Jimenez-Useche *et al*.^33^ later concluded that DNA methylation makes the nucleosomal DNA ends less compact.

**Figure 3:**
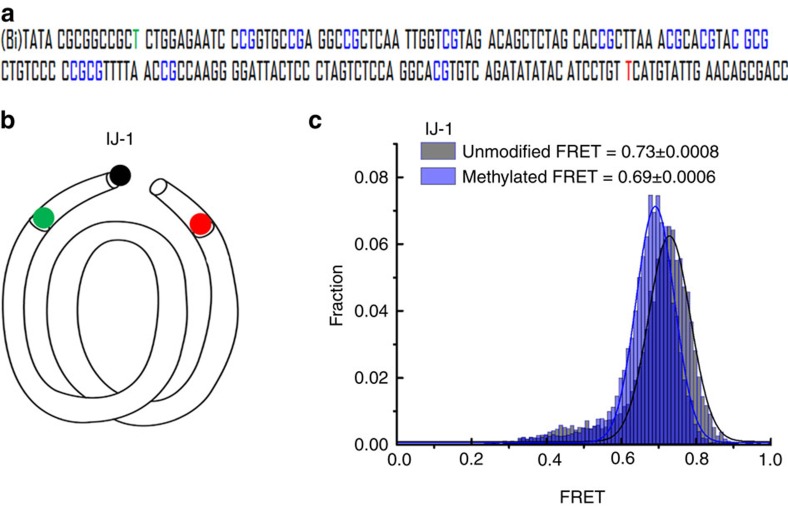
5-mC loosens packing of nucleosomal DNA ends. (**a**) A DNA construct containing the 601 sequence used to reconstitute a nucleosome. CpG sites methylated using the M.SssI enzyme are highlighted in blue. The nucleotides carrying the Cy3 and Cy5 fluorophores are shown in green and red, respectively. (**b**) Schematic representation of a nucleosomal DNA with the IJ-1 labelling position. (**c**) smFRET histogram of IJ-1 nucleosomes with and without methylation. Gaussian fits and the average FRET values are shown.

Here we used smFRET to examine the effect of DNA methylation on a nucleosome formed on the 601 sequence, which can position the nucleosome precisely without translational ambiguity and has been used for previous high-resolution single-molecule studies (Fig. 3a)^29^,^31^,^32^,^33^,^36^,^41^,^42^,^43^,^44^,^45^,^46^,^47^,^48^,^49^,^50^,^51^,^52^,^53^. We attached a pair of donor and acceptor fluorophores at the first nucleotides just outside of the 601 core sequence (Fig. 3b). The DNA constructs were made by PCR amplification using labelled primers and methylated enzymatically using *Spiroplasma* methyltransferase M.SssI (refs 33, 34). The completeness of methylations was confirmed by digestion assay using a restriction enzyme BstUI that does not function on 5-mC (Supplementary Fig. 5). Nucleosomes were reconstituted from the unmodified or methylated DNA constructs and histone octamers from *Xenopus laveis*.

Nucleosomes were immobilized on a PEG-coated quartz slide. In agreement with well-defined positioning of the nucleosome indicated by electrophoretic mobility shift assay (Supplementary Fig. 5), smFRET efficiency histogram showed a single-peaked distribution (Fig. 3c). Nucleosomes with methylated DNA showed a lower FRET efficiency (0.69±0.006) than unmodified nucleosomes (0.73±0.008). The reduction in FRET indicates that DNA methylation causes loosening of the nucleosomal DNA ends, supporting the study by Jimenez-Useche *et. al.*^33^ but contradicting the study by Choy *et. al.*^34^ While investigating this discrepancy we found that having the methyltransferase present during the measurement, as was the case in Choy *et al*., induces FRET increases to 0.83, likely due to the binding of the enzyme to the nucleosome. Indeed, after a 90-min incubation, the FRET value was found to return to the original lower level, likely due to enzyme dissociation (Supplementary Fig. 6). In our experiments, methylated DNA was purified before nucleosome reconstitution thus ensuring that the nucleosome is free of methyltransferase. Overall, our data are consistent with the notion that spontaneous unwrapping of DNA ends from the histone core is enhanced on DNA methylation. Because wrapping/unwrapping dynamics occur at the millisecond timescale^32^ the effect of methylation is seen as a decrease in time-averaged FRET.

### Assay for probing nucleosome mechanical stability

In an earlier work, we demonstrated that mechanical stability of a nucleosome is controlled by DNA flexibility^36^, suggesting that DNA flexibility can be used as a proxy of nucleosome stability. If the DNA is more flexible, the sharply bent DNA conformation in a nucleosome is better tolerated, even when DNA is under tension, and vice versa. Here we showed that 5-mC decreases DNA flexibility, whereas 5-fC increases it. Therefore, we sought to examine how 5-mC and 5-fC change the nucleosome's mechanical stability by measuring conformational dynamics using smFRET as a function of an external force applied by optical tweezers^54^,^55^.

**Figure 4:**
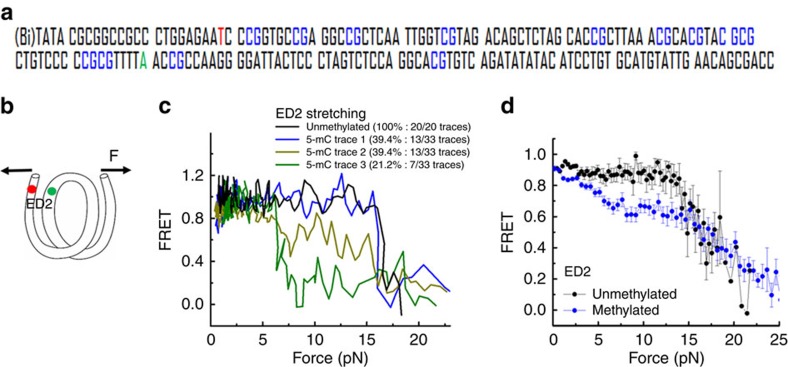
5-mC reduces initial unwrapping force of nucleosome on the strong side. (**a**) The DNA construct containing the 601 sequence used to reconstitute nucleosome. CpG sites methylated using M.SssI enzyme are show in blue. The nucleotides carrying the Cy3 and Cy5 fluorophores are shown in green and red, respectively. (**b**) Schematic representation of a nucleosomal DNA with the ED2 labelling scheme used for unwrapping experiments. Arrows stand for the force applied to the ends of DNA with a magnitude of **F**. (**c**) Representative single-molecule stretching traces of an ED2 nucleosome with unmodified and methylated DNA. (**d**) Averaging of stretching traces for the ED2 nucleosomes with unmodified (20 traces) and methylated (33 traces) DNA.

In the fluorescence-force spectroscopy assay, a nucleosome was anchored to a PEG-coated glass surface via a biotin–neutravidin pair on one end of the DNA, and was pulled by an optical trap via a λ-DNA tethered to the other end^36^. We attached a pair of donor and acceptor fluorophores to the DNA to probe unwrapping of nucleosomal DNA. Typically, we increased the force gradually from a low value (typically between 0.4 and 1.0 pN) to a predetermined higher value, and then gradually decreased the force back to the low value. As the force increases, we observe a reduction in FRET indicating unwrapping of nucleosomal DNA (Fig. 4c,d). As a measure of the nucleosome's mechanical stability, we used the force range, where the largest drop in FRET efficiency occurs.

### 5-mC destabilizes nucleosome mechanically

In our earlier work, we observed that two DNA ends of the 601 nucleosome unwrap asymmetrically—one end unwraps at lower forces (∼3 pN) than the other end (∼15 pN)^36^. Here we chose to probe the effect of DNA methylation using the labelling configuration called ED2, which reports on DNA unwrapping of the strong side of the nucleosome^36^, for the following reasons. (1) Because nucleosomes with the more flexible DNA can withstand the stronger unwrapping forces^36^ we anticipate DNA methylation, which we have shown here to make DNA less flexible, would make nucleosomes unwrap at lower forces. Such a reduction in mechanical stability can be probed with a higher contrast at the strong side of the nucleosome. (2) The strong half of the 601 sequence contains 18 methylation sites, whereas the weak half contains only 8 (Fig. 4a). In ED2, a donor fluorophore was incorporated to the nucleotide 58 from the right entry of the 601 sequence on the bottom strand (J58), and the acceptor was attached to the nucleotide 9 from the left entry of the 601 sequence on the top strand (I9) (Fig. 4a,b).

For the unmodified construct, all stretching traces, that is, the dependence of the FRET efficiency on gradually increasing force applied to the ends of the nucleosomal DNA, showed the same pattern of stable FRET at low forces and a drop at high forces (∼15 pN; Fig. 4c). Subsequent pulls of the same molecule showed the same pattern, indicating the absence of an irreversible change. For the fully methylated construct, although the FRET values at the lowest forces matched those from the unmodified construct, we observed diverse behaviours on force increases. In all 39.4% of traces (13 of 33) showed the same behaviour as the unmodified nucleosomes, staying in high FRET until a sudden drop at high forces; 39.4% of traces (13 of 33) showed an additional gradual decrease before the final drop occurred at ∼15 pN; and 21.2% of traces (7 of 33) showed a major drop in FRET at low force range (∼5 pN; Fig. 4c). Averaging FRET values as a function of force for all traces (Fig. 4d) showed a pronounced decrease in the low force range caused by methylation, indicating that 5-mC assists the early unwrapping of the DNA termini. In the high force range, the slopes at forces higher than 15 pN were similar for both constructs, implying that the final stage of inner turn unwrapping is not affected by DNA methylation.

### 5-fC enhances nucleosome mechanical stability

**Figure 5:**
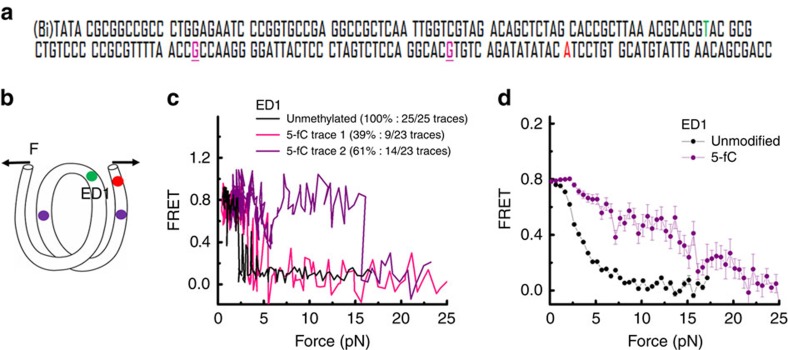
5-fC enhances nucleosome stability. (**a**) The DNA construct containing the 601 sequence used to reconstitute nucleosome. CpG sites with 5-fC modifications are shown in purple and underlined. The nucleotides carrying the Cy3 and Cy5 fluorophores are shown in green and red, respectively. (**b**) Schematic representation of a nucleosomal DNA, with the ED1 labelling scheme containing two copies of 5-fC at nucleotide 22 (J22) and 54 (J54) of the bottom strand of the 601 sequence. 5-fC is shown in purple, Cy3 and Cy5 are shown in green and red, respectively. (**c**) Representative stretching traces of an ED1 nucleosome with unmodified DNA (black) and DNA containing two copies of 5-fC (red and purple). (**d**) Averaging of the stretching traces for the ED1 nucleosomes with unmodified DNA (25 traces, shown in black) and DNA containing two copies of 5-fC (23 traces, shown in purple).

Next, we investigated the effect of 5-fC on the mechanical stability of the nucleosome. Because 5-fC enhances DNA flexibility even when a very small number of modifications is present, we expected 5-fC modifications to make binding of DNA to the histone core stronger, and therefore chose to examine the mechanical stability of the weak side. A donor fluorophore was incorporated to the nucleotide 68 from the left entry of the 601 sequence on the top strand (I68), and the acceptor was attached to the nucleotide 7 from the right entry of the 601 sequence on the bottom strand (J7) (Fig. 5a,b). This labelling scheme was previously used to probe unwrapping of the weak side^36^ (ED1 labelling scheme—Fig. 5b). We introduced one 5-fC in the middle region of the outer turn of the weak side and another in the middle region of the inner turn of the weak side (Fig. 5a). The unmodified and the 5-fC constructs yielded nucleosomes of the same electromobility, indicating that 5-fC does not perturb nucleosome positioning (Supplementary Fig. 7).

As previously reported^36^, all of the stretching traces of the weak side of the unmodified construct probed by ED1 displayed a gradual decrease in FRET as the force increased followed by fast fluctuations, and finally a sharp decrease at ∼3–5 pN (Fig. 5c). For the 5-fC construct, although 39% of traces (9 of 23) exhibited similar stretching patterns as in the case of the unmodified nucleosome, a majority, 61% (14 of 23), showed the final drop in FRET at higher force. The averaged FRET-versus-force pattern clearly showed an increase in unwrapping force for the 5-fC-containing nucleosomes (Fig. 5d). Therefore, even a very small number of 5-fC in a nucleosome can greatly increase its mechanical stability.

## Discussion

Here we report a comprehensive survey of the effect of cytosine modifications on DNA flexibility. Our observation of the reduction of DNA flexibility by 5-mC is consistent with previous studies using nuclear magnetic resonance^22^,^56^, DNA cyclization assay^23^ and DNA transportation through a synthetic nanopore^57^. Methylation-induced increases in DNA stiffness likely arise from the restriction of the conformational fluctuations caused by the bulky methyl groups^22^,^24^.

Though *in vivo* 5-fC level is low^11^,^19^, the recent studies indicate that a large portion of 5-fC inside mammalian cells tend to be stable, suggesting additional roles of 5-fC as a potential stable mark beyond being a demethylation intermediate^58^. In addition, structural studies revealed a unique property of 5-fC in distorting DNA duplex when a cluster of 5-fCs are presented^58^. We showed here that 5-fC can have a disproportionately large effect on DNA flexibility and nucleosome stability because even a single copy of 5-fC within 90 bp of DNA can increase the DNA cyclization rate by threefold. The significant enhancement of DNA flexibility induced by 5-fC may assist a substrate recognition mechanism for thymine DNA glycosylase (TDG), a base excision enzyme that works most efficiently on 5-fC substrate in the demethylation pathway. TDG shares the base-flipping mechanism of glycosylases family to remove a base, a process that requires disruption of base stacking and formation of a sharp kink^25^. Interestingly, 5-fC is the most favourite substrate for TDG^6^, possibly because 5-fC reduces the energy required for DNA bending and unstacking during base-flipping by TDG, similar to the recognition mechanism of other DNA repair proteins^25^.

Cloutier and Widom^59^ previously reported spontaneous cyclization of 120-bp DNA molecules, suggesting that local kinking could facilitate the extreme bendability of such short DNA fragments. Here our molecular dynamics simulations demonstrate that cytosine modifications affect the fluctuations of the structural parameters (rolls, twist, slide, shear, stretch, stagger, buckle, propeller and opening) without significantly changing their average values. Such changes in the fluctuations of the structural parameters explain acceleration of DNA cyclization by 5-fC and 5-hmC, and suppression of DNA looping by 5-mC. This result also indicates that, in probing the effect of cytosine modifications, our single-molecule cyclization assay reports mainly on DNA dynamic flexibility rather than on static bending and other deformations.

*In vivo*, methylated cytosine correlated with gene inactivation. The three possible mechanisms for negative gene regulation of methylated cytosine are as follows: (1) stabilizations of the chromatin at the level of single nucleosomes; (2) facilitation of chromatin packaging into higher-order structures; and (3) preventions of transcriptional activator binding or facilitations of transcriptional repressor bindings. We found that methylated cytosine loosens packaging of the DNA ends of an isolated nucleosome and makes the nucleosome unwrap at a lower force, which by itself would not make it more difficult to gain access to the nucleosomal DNA. Therefore, we suggest that transcription suppression role of DNA methylation may be mediated mainly through the latter two mechanisms described above. For example, loosening of the DNA end may allow histone tail binding to the adjacent nucleosomes and mediation of inter-nucleosomes interaction, which facilitates packaging of chromatin into higher-order structures^60^,^61^.

The presence of two copies of 5-fC in 147 bp (that is, 0.68%) of DNA of the 601 sequence results in a remarkable enhancement of nucleosome mechanical stability. This observation together with the reduced mechanical stability of nucleosomes containing many copies of 5-mC gives further support to our previously reported correlation between DNA flexibility and nucleosome stability: the more flexible DNA is, the more stably it is bound to histone octamer core and vice versa^36^. The increased nucleosome stability induced by 5-fC may explain enrichment of 5-fC at poised enhancers and the transcription start sites (TSSs) of low-expression genes^19^: enhancement of nucleosome stability limits exposure of nucleosomal DNA to transcription machinery at enhancers and transcription start sites.

## Methods

### Oligo synthesis

Unmodified and 5-mC phosphoramidites were purchased from Glen Research. 5-fC and 5-caC phosphoramidites and DNA oligos containing them were prepared by following our published procedure, and 5-hmC oligos were obtained directly from 5-fC oligos by treatment with sodium borohydride^62^. All the DNA oligos were purified by HPLC with a C18 reverse-phase column.

### Preparation of DNA constructs

DNA constructs were prepared by ligation or PCR. In ligation method, each strand of DNA constructs was prepared by ligation of synthesized DNA fragments containing modifications, and purified using 14% denaturing PAGE gel. The two purified complement strands were annealed by heating to 90 °C followed by slow cooling over 3–4 h. For cyclization measurements, the DNA construct is an 80-bp DNA fragment containing modifications as indicated in Fig. 1 and, two 10-nucleotide-long 5′ overhangs with Cy3 and Cy5 at 5′-ends that were complementary to each other. Biotin was incorporated 20 bp apart from the 5′-end of the bottom strand. For nucleosome measurements, the DNA construct contained the 601 sequence flanked by a 14-bp spacer to biotin for surface tethering and a 20-bp spacer connecting to a 12-nucleotide overhang that was used for annealing to λ-DNA. Method for preparing DNA construct by PCR and positions of dyes were listed in our previous publication^36^.

### Nucleosome reconstitution

The 601 DNA templates were reconstituted with *X. laevis* recombinant histone octamer (purchased from Colorado State University) by salt dialysis^63^. Reconstituted nucleosomes were stored at 4 °C in the dark typically at concentrations of 100–200 nM, and used within 2 weeks. The efficiency of nucleosome reconstitution was measured by 5% native PAGE gel electrophoresis.

### Single-molecule DNA cyclization measurement

We recently developed a single-molecule DNA cyclization assay to quantify the flexibility of a short double-stranded DNAs (<100 bp)^38^. DNA fragments for cyclization measurement were immobilized on a PEG-coated microscope slide via biotin–neutravidin linkage. After introducing a high-salt-buffered solution (20 mM Tris-HCl (pH 8.0), 1 M NaCl, 0.5% w/v D-glucose (Sigma), 165 U ml^−1^ glucose oxidase (Sigma), 2,170 U ml^−1^ catalase (Roche) and 3 mM Trolox (Sigma)), annealing of the two overhangs was detected as a FRET increase. We determined the fraction of high-FRET population corresponding to the looped molecules as a function of time.

### Single-molecule FRET experiments of nucleosomes

The nucleosome sample was immobilized on a PEG (mixture of mPEG-SVA and Biotin-PEG-SVA, Laysan Bio) coated slide at 50 pM nucleosome dilution buffer (10 mM Tris-HCl (pH 8.0), 50 mM NaCl and 1 mM MgCl_2_) through a biotin–neutravidin linker. Single-molecule FRET data were taken in the imaging buffer (50 mM Tris-HCl (pH 8.0), 50 mM NaCl, 1 mM MgCl_2_, 0.5 mg ml^−1^ BSA (NEB), 0.5% w/v D-glucose (Sigma), 165 U ml^−1^ glucose oxidase (Sigma), 2,170 U ml^−1^ catalase (Roche) and 3 mM Trolox (Sigma)) using a home-build total internal reflection fluorescence microscope.

### Nucleosome-unwrapping measurements

The nucleosome was annealed to λ-DNA and an oligonucleotide containing digoxigenin at the final concentration of 8 nM. Samples were stored at 4 °C in the dark. On the day of measurement, nucleosome solution was diluted to 10 pM in a dilution buffer (10 mM Tris-HCl (pH 8.0), 50 mM NaCl and 1 mM MgCl_2_) and immobilized on a PEG-coated microscope slide. To attach beads to the free end of the λ-DNA tether, 1 μm anti-digoxigenin-coated polystyrene beads (Polysciences) diluted in nucleosome dilution buffer were added to the imaging chamber for about 30 min. Finally, force-fluorescence data acquisition was performed in the imaging buffer (50 mM Tris-HCl (pH 8.0), 50 mM NaCl, 1 mM MgCl_2_, 0.5 mg ml^−1^ BSA (NEB), 0.5 mg ml^−1^ tRNA (Ambion), 0.1% v/v Tween-20 (Sigma), 0.5% w/v D-glucose (Sigma), 165 U ml^−1^ glucose oxidase (Sigma), 2,170 U ml^−1^ catalase (Roche) and 3 mM Trolox (Sigma)).

Data acquisition was performed using a home-built set-up^54^. First, the origin of a tether connected to the trapped bead was determined by stretching the tether in two opposite directions along the *x* and *y* axes. Then, the fluorescence spot on the tether was determined by scanning the confocal laser after separating the trapped bead from its origin by 14 μm. The nucleosome was stretched and relaxed by moving the stage between 14 and 16.8–17.2 μm at the speed of 455 nm s^−1^. Fluorescence emission was detected for 20 ms after each step in stage movement, by scanning the confocal excitation concurrently with the stage movement.

All single-molecule measurements were performed at 22 °C.

### Molecular dynamics simulations

All molecular dynamics simulations were performed using the NAMD programme^64^, the CHARMM36 force field^65^ and custom sodium–phosphate interaction parameters^66^. Parameters for the formyl- and hydroxymethylcytosine bases were derived using the CGenFF web server^67^. The van der Waals and short-range electrostatic energies were calculated using a 10–12-Å switching scheme. The long-range electrostatic interactions were computed using the particle-mesh Ewald scheme and the grid size of 1.2 Å (ref. 68). The integration time step was 2 fs; 2–2–6 fs multiple time stepping was used. All simulations were performed under periodic boundary conditions, constant temperature (298 K) and constant pressure (1 bar).

The initial simulation system was built by using the 3D-DART web server^69^, to produce an idealized all-atom model of B-DNA containing no chemical modifications. The DNA fragment was 70-bp long and had the sequence of the central region of the experimental construct: 5′-TACCTCAATATAGACTCCCTCCGGTGCCGAGGCCGCTCAATTGGTCGTAGGACTATCCTCACCTCCACCG-3′. Four additional structures containing the 5-fC, 5-mC, 5-hmC or 5-caC modifications in eight cytosine nucleotides at four CpG steps were built based on the unmodified B-DNA structure using the CHARMM programme^70^. Each of the four DNA molecules was submerged in a rectangular volume of 1-M NaCl solution measuring ∼100 × 70 × 260 Å^3^. The solvated systems were energy-minimized for 4,800 steps and equilibrated for about 5 ns having all heavy atoms restrained to their original coordinates using harmonic potentials (*k*=1 kcal mol^−1^ Å^−2^). Following that, each system was simulated for ∼250 ns. Using coordinates that were saved every 4.8 ps, structural parameters of DNA were computed using the 3DNA programme^39^. For error bars in Fig. 5 and Supplementary Figs 2 and 3, first we computed the s.e.'s of the 3DNA parameters for each base-pair step or each base pair treating a 20-ns block average as an independent measurement. Then, the s.e.'s were evaluated using the conventional error propagation rule.

## Additional information

**How to cite this article:** Ngo, T. T. M. *et al*. Effects of cytosine modifications on DNA flexibility and nucleosome mechanical stability. *Nat. Commun.* 7:10813 doi: 10.1038/ncomms10813 (2016).

## Supplementary Material

Supplementary Information Supplementary Figures 1-7

## References

[b1] Law J. A. & Jacobsen S. E. Establishing, maintaining and modifying DNA methylation patterns in plants and animals. Nat. Rev. Genet. 11, 204–220 (2010). 20142834 10.1038/nrg2719 PMC3034103

[b2] Jaenisch R. & Bird A. Epigenetic regulation of gene expression: how the genome integrates intrinsic and environmental signals. Nat. Genet. 33, 245–254 (2003). 12610534 10.1038/ng1089

[b3] Smith Z. D. & Meissner A. DNA methylation: roles in mammalian development. Nat. Rev. Genet. 14, 204–220 (2013). 23400093 10.1038/nrg3354

[b4] Hajkova P. . Epigenetic reprogramming in mouse primordial germ cells. Mech. Dev. 117, 15–23 (2002). 12204247 10.1016/s0925-4773(02)00181-8

[b5] Mayer W., Niveleau A., Walter J., Fundele R. & Haaf T. Embryogenesis—demethylation of the zygotic paternal genome. Nature 403, 501–502 (2000). 10676950 10.1038/35000656

[b6] Wu H. & Zhang Y. Reversing DNA methylation: mechanisms, genomics, and biological functions. Cell 156, 45–68 (2014). 24439369 10.1016/j.cell.2013.12.019 PMC3938284

[b7] Kohli R. M. & Zhang Y. TET enzymes, TDG and the dynamics of DNA demethylation. Nature 502, 472–479 (2013). 24153300 10.1038/nature12750 PMC4046508

[b8] Pastor W. A., Aravind L. & Rao A. TETonic shift: biological roles of TET proteins in DNA demethylation and transcription. Nat. Rev. Mol. Cell Biol. 14, 341–356 (2013). 23698584 10.1038/nrm3589 PMC3804139

[b9] Kriaucionis S. & Heintz N. The nuclear DNA base 5-hydroxymethylcytosine is present in Purkinje neurons and the brain. Science 324, 929–930 (2009). 19372393 10.1126/science.1169786 PMC3263819

[b10] Tahiliani M. . Conversion of 5-methylcytosine to 5-hydroxymethylcytosine in mammalian DNA by MLL partner TET1. Science 324, 930–935 (2009). 19372391 10.1126/science.1170116 PMC2715015

[b11] Pfaffeneder T. . The discovery of 5-formylcytosine in embryonic stem cell DNA. Angew. Chem. Int. Ed. Engl. 50, 7008–7012 (2011). 21721093 10.1002/anie.201103899

[b12] Ito S. . Tet proteins can convert 5-methylcytosine to 5-formylcytosine and 5-carboxylcytosine. Science 333, 1300–1303 (2011). 21778364 10.1126/science.1210597 PMC3495246

[b13] He Y. F. . Tet-mediated formation of 5-carboxylcytosine and its excision by TDG in mammalian DNA. Science 333, 1303–1307 (2011). 21817016 10.1126/science.1210944 PMC3462231

[b14] Leonhardt H., Rahn H. P. & Cardoso M. C. Functional links between nuclear structure, gene expression, DNA replication, and methylation. Crit. Rev. Eukaryot. Gene Expr. 9, 345–351 (1999). 10651251 10.1615/critreveukargeneexpr.v9.i3-4.190

[b15] Pastor W. A. . Genome-wide mapping of 5-hydroxymethylcytosine in embryonic stem cells. Nature 473, 394–397 (2011). 21552279 10.1038/nature10102 PMC3124347

[b16] Szulwach K. E. . Integrating 5-hydroxymethylcytosine into the Epigenomic Landscape of Human Embryonic Stem Cells. PLoS Genet. 7, e1002154 (2011). 21731508 10.1371/journal.pgen.1002154 PMC3121778

[b17] Wu H. . Genome-wide analysis of 5-hydroxymethylcytosine distribution reveals its dual function in transcriptional regulation in mouse embryonic stem cells. Genes Dev. 25, 679–684 (2011). 21460036 10.1101/gad.2036011 PMC3070931

[b18] Xu Y. . Genome-wide regulation of 5hmC, 5mC, and gene expression by Tet1 hydroxylase in mouse embryonic stem cells. Mol. Cell 42, 451–464 (2011). 21514197 10.1016/j.molcel.2011.04.005 PMC3099128

[b19] Song C.-X. . Genome-wide profiling of 5-formylcytosine reveals its roles in epigenetic priming. Cell 153, 678–691 (2013). 23602153 10.1016/j.cell.2013.04.001 PMC3657391

[b20] Lu X. . Base-resolution maps of 5-formylcytosine and 5-carboxylcytosine reveal genome-wide DNA demethylation dynamics. Cell Res. 25, 386–389 (2015). 25591929 10.1038/cr.2015.5 PMC4349244

[b21] Tate P. H. & Bird A. P. Effects of DNA methylation on DNA-binding proteins and gene expression. Curr. Opin. Genet. Dev. 3, 226–231 (1993). 8504247 10.1016/0959-437x(93)90027-m

[b22] Derreumaux S., Chaoui M., Tevanian G. & Fermandjian S. Impact of CpG methylation on structure, dynamics and solvation of cAMP DNA responsive element. Nucleic Acids Res. 29, 2314–2326 (2001). 11376150 10.1093/nar/29.11.2314 PMC55717

[b23] Nathan D. & Crothers D. M. Bending and flexibility of methylated and unmethylated EcoRI DNA. J. Mol. Biol. 316, 7–17 (2002). 11829499 10.1006/jmbi.2001.5247

[b24] Perez A. . Impact of methylation on the physical properties of DNA. Biophys. J. 102, 2140–2148 (2012). 22824278 10.1016/j.bpj.2012.03.056 PMC3341543

[b25] Yang W. Structure and mechanism for DNA lesion recognition. Cell Res. 18, 184–197 (2008). 18157156 10.1038/cr.2007.116

[b26] Wanunu M. . Discrimination of methylcytosine from hydroxymethylcytosine in DNA molecules. J. Am. Chem. Soc. 133, 486–492 (2011). 21155562 10.1021/ja107836t PMC3081508

[b27] Luger K., Mader A. W., Richmond R. K., Sargent D. F. & Richmond T. J. Crystal structure of the nucleosome core particle at 2.8 angstrom resolution. Nature 389, 251–260 (1997). 9305837 10.1038/38444

[b28] Churchman L. S. & Weissman J. S. Nascent transcript sequencing visualizes transcription at nucleotide resolution. Nature 469, 368–373 (2011). 21248844 10.1038/nature09652 PMC3880149

[b29] Bondarenko V. A. . Nucleosomes can form a polar barrier to transcript elongation by RNA polymerase II. Mol. Cell 24, 469–479 (2006). 17081995 10.1016/j.molcel.2006.09.009

[b30] Gorman J., Plys A. J., Visnapuu M.-L., Alani E. & Greene E. C. Visualizing one-dimensional diffusion of eukaryotic DNA repair factors along a chromatin lattice. Nat. Struct. Mol. Biol. 17, 932–U937 (2010). 20657586 10.1038/nsmb.1858 PMC2953804

[b31] Hodges C., Bintu L., Lubkowska L., Kashlev M. & Bustamante C. Nucleosomal fluctuations govern the transcription dynamics of RNA polymerase II. Science 325, 626–628 (2009). 19644123 10.1126/science.1172926 PMC2775800

[b32] Li G. & Widom J. Nucleosomes facilitate their own invasion. Nat. Struct. Mol. Biol. 11, 763–769 (2004). 15258568 10.1038/nsmb801

[b33] Jimenez-Useche I. . DNA methylation regulated nucleosome dynamics. Sci. Rep. 3, 1–5 (2013). 10.1038/srep02121 PMC3698496 23817195

[b34] Choy J. S. . DNA methylation increases nucleosome compaction and rigidity. J. Am. Chem. Soc. 132, 1782–1783 (2010). 20095602 10.1021/ja910264z PMC4167393

[b35] Lee J. Y. & Lee T.-H. Effects of DNA methylation on the structure of nucleosomes. J. Am. Chem. Soc. 134, 173–175 (2012). 22148575 10.1021/ja210273w PMC3257366

[b36] Ngo T. T., Zhang Q., Zhou R., Yodh J. G. & Ha T. Asymmetric unwrapping of nucleosomes under tension directed by DNA local flexibility. Cell 160, 1135–1144 (2015). 25768909 10.1016/j.cell.2015.02.001 PMC4409768

[b37] Shore D., Langowski J. & Baldwin R. L. DNA flexibility studied by covalent closure of short fragments into circles. Proc. Natl Acad. Sci. USA 78, 4833–4837 (1981). 6272277 10.1073/pnas.78.8.4833 PMC320266

[b38] Vafabakhsh R. & Ha T. Extreme bendability of DNA less than 100 base pairs long revealed by single-molecule cyclization. Science 337, 1097–1101 (2012). 22936778 10.1126/science.1224139 PMC3565842

[b39] Lu X. J. & Olson W. K. 3DNA: a software package for the analysis, rebuilding and visualization of three-dimensional nucleic acid structures. Nucleic Acids Res. 31, 5108–5121 (2003). 12930962 10.1093/nar/gkg680 PMC212791

[b40] Czapla L., Swigon D. & Olson W. K. Sequence-dependent effects in the cyclization of short DNA. J. Chem. Theory Comput. 2, 685–695 (2006). 26626674 10.1021/ct060025+

[b41] Bintu L. . Nucleosomal elements that control the topography of the barrier to transcription. Cell 151, 738–749 (2012). 23141536 10.1016/j.cell.2012.10.009 PMC3508686

[b42] Bintu L. . The elongation rate of RNA polymerase determines the fate of transcribed nucleosomes. Nat. Struct. Mol. Biol. 18, 1394–1399 (2011). 22081017 10.1038/nsmb.2164 PMC3279329

[b43] Boehm V. . Nucleosome accessibility governed by the dimer/tetramer interface. Nucleic Acids Res. 39, 3093–3102 (2011). 21177647 10.1093/nar/gkq1279 PMC3082900

[b44] Brower-Toland B. D. . Mechanical disruption of individual nucleosomes reveals a reversible multistage release of DNA. Proc. Natl Acad. Sci. USA 99, 1960–1965 (2002). 11854495 10.1073/pnas.022638399 PMC122302

[b45] Buning R. & van Noort J. Single-pair FRET experiments on nucleosome conformational dynamics. Biochimie 92, 1729–1740 (2010). 20800089 10.1016/j.biochi.2010.08.010

[b46] Deindl S. . ISWI remodelers slide nucleosomes with coordinated multi-base-pair entry steps and single-base-pair exit steps. Cell 152, 442–452 (2013). 23374341 10.1016/j.cell.2012.12.040 PMC3647478

[b47] Hall M. A. . High-resolution dynamic mapping of histone-DNA interactions in a nucleosome. Nat. Struct. Mol. Biol. 16, 124–129 (2009). 19136959 10.1038/nsmb.1526 PMC2635915

[b48] Kruithof M. & van Noort J. Hidden Markov analysis of nucleosome unwrapping under force. Biophys. J. 96, 3708–3715 (2009). 19413976 10.1016/j.bpj.2009.01.048 PMC2711431

[b49] Li G., Levitus M., Bustamante C. & Widom J. Rapid spontaneous accessibility of nucleosomal DNA. Nat. Struct. Mol. Biol. 12, 46–53 (2005). 15580276 10.1038/nsmb869

[b50] Makde R. D., England J. R., Yennawar H. P. & Tan S. Structure of RCC1 chromatin factor bound to the nucleosome core particle. Nature 467, 562–566 (2010). 20739938 10.1038/nature09321 PMC3168546

[b51] Mihardja S., Spakowitz A. J., Zhang Y. & Bustamante C. Effect of force on mononucleosomal dynamics. Proc. Natl Acad. Sci. USA 103, 15871–15876 (2006). 17043216 10.1073/pnas.0607526103 PMC1635095

[b52] North J. A. . Regulation of the nucleosome unwrapping rate controls DNA accessibility. Nucleic Acids Res. 40, 10215–10227 (2012). 22965129 10.1093/nar/gks747 PMC3488218

[b53] Shundrovsky A., Smith C. L., Lis J. T., Peterson C. L. & Wang M. D. Probing SWI/SNF remodeling of the nucleosome by unzipping single DNA molecules. Nat. Struct. Mol. Biol. 13, 549–554 (2006). 16732285 10.1038/nsmb1102

[b54] Hohng S. . Fluorescence-force spectroscopy maps two-dimensional reaction landscape of the Holliday junction. Science 318, 279–283 (2007). 17932299 10.1126/science.1146113 PMC3558530

[b55] Zhou R. . SSB functions as a sliding platform that migrates on DNA via reptation. Cell 146, 222–232 (2011). 21784244 10.1016/j.cell.2011.06.036 PMC3155616

[b56] Geahigan K. B., Meints G. A., Hatcher M. E., Orban J. & Drobny G. P. The dynamic impact of CpG methylation in DNA. Biochemistry 39, 4939–4946 (2000). 10769153 10.1021/bi9917636

[b57] Mirsaidov U. . Nanoelectromechanics of methylated DNA in a synthetic nanopore. Biophys. J. 96, L32–L34 (2009). 19217843 10.1016/j.bpj.2008.12.3760 PMC2717226

[b58] Bachman M. . 5-Formylcytosine can be a stable DNA modification in mammals. Nat. Chem. Biol. 11, 555–557 (2015). 26098680 10.1038/nchembio.1848 PMC5486442

[b59] Cloutier T. E. & Widom J. Spontaneous sharp bending of double-stranded DNA. Mol. Cell 14, 355–362 (2004). 15125838 10.1016/s1097-2765(04)00210-2

[b60] Bednar J. . Nucleosomes, linker DNA, and linker histone form a unique structural motif that directs the higher-order folding and compaction of chromatin. Proc. Natl Acad. Sci. USA 95, 14173–14178 (1998). 9826673 10.1073/pnas.95.24.14173 PMC24346

[b61] Pepenella S., Murphy K. J. & Hayes J. J. Intra- and inter-nucleosome interactions of the core histone tail domains in higher-order chromatin structure. Chromosoma 123, 3–13 (2014). 23996014 10.1007/s00412-013-0435-8 PMC3938996

[b62] Dai Q. & He C. Syntheses of 5-formyl- and 5-carboxyl-dC containing DNA oligos as potential oxidation products of 5-hydroxymethylcytosine in DNA. Org. Lett. 13, 3446–3449 (2011). 21648398 10.1021/ol201189n PMC3150843

[b63] Ha T. . Probing the interaction between two single molecules: fluorescence resonance energy transfer between a single donor and a single acceptor. Proc. Natl Acad. Sci. USA 93, 6264–6268 (1996). 8692803 10.1073/pnas.93.13.6264 PMC39010

[b64] Phillips J. C. . Scalable molecular dynamics with NAMD. J. Comput. Chem. 26, 1781–1802 (2005). 16222654 10.1002/jcc.20289 PMC2486339

[b65] Hart K. . Optimization of the CHARMM Additive force field for DNA: improved treatment of the BI/BII conformational equilibrium. J. Chem. Theory Comput. 8, 348–362 (2012). 22368531 10.1021/ct200723y PMC3285246

[b66] Yoo J. J. & Aksimentiev A. Improved parametrization of Li+, Na+, K+, and Mg2+ ions for all-atom molecular dynamics simulations of nucleic acid systems. J. Phys. Chem. Lett. 3, 45–50 (2012).

[b67] Vanommeslaeghe K. . CHARMM general force field: a force field for drug-like molecules compatible with the CHARMM all-atom additive biological force fields. J. Comput. Chem. 31, 671–690 (2010). 19575467 10.1002/jcc.21367 PMC2888302

[b68] Darden T., York D. & Pedersen L. Particle mesh Ewald: an *N* log (*N*) method for Ewald sums in large systems. J. Chem. Phys. 98, 10089–10092 (1993).

[b69] van Dijk M. & Bonvin A. 3D-DART: a DNA structure modelling server. Nucleic Acids Res. 37, W235–W239 (2009). 19417072 10.1093/nar/gkp287 PMC2703913

[b70] Brooks B. R. . CHARMM—a program for macromolecular energy, minimization, and dynamics calculations. J. Comput. Chem. 4, 187–217 (1983).

