# Supplementary material for: Effects of cytosine modifications on DNA flexibility and nucleosome mechanical stability
Source: Nat Commun. 2016 Feb 24;7:10813. doi: 10.1038/ncomms10813 (PMC4770088; doi:10.1038/ncomms10813)
Supplement: Supplementary Information — Supplementary Figures 1-7 [file ncomms10813-s1.pdf]

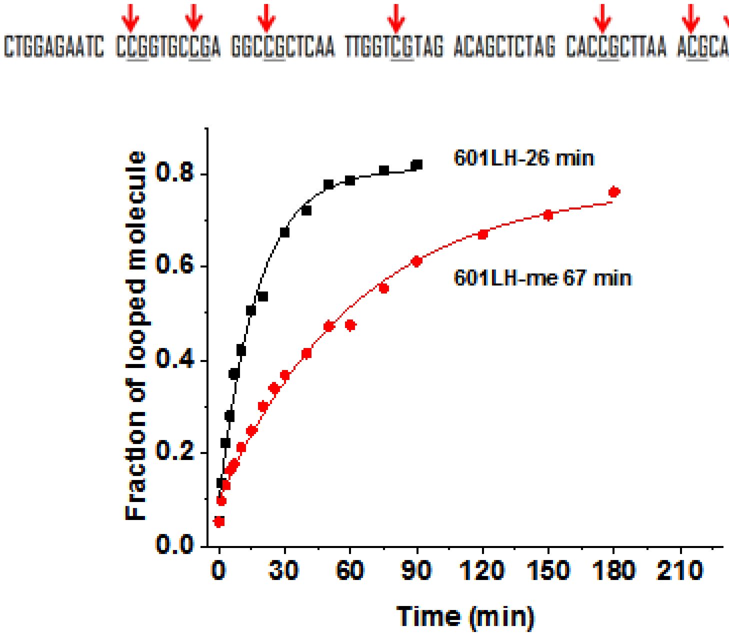

Supplementary Figure 1: Cyclization measurement of the unmodified and enzymatically methylated left half of the 601 sequence. CpG sites which were methylated using M.SssI enzyme are underlined and marked by red arrows.

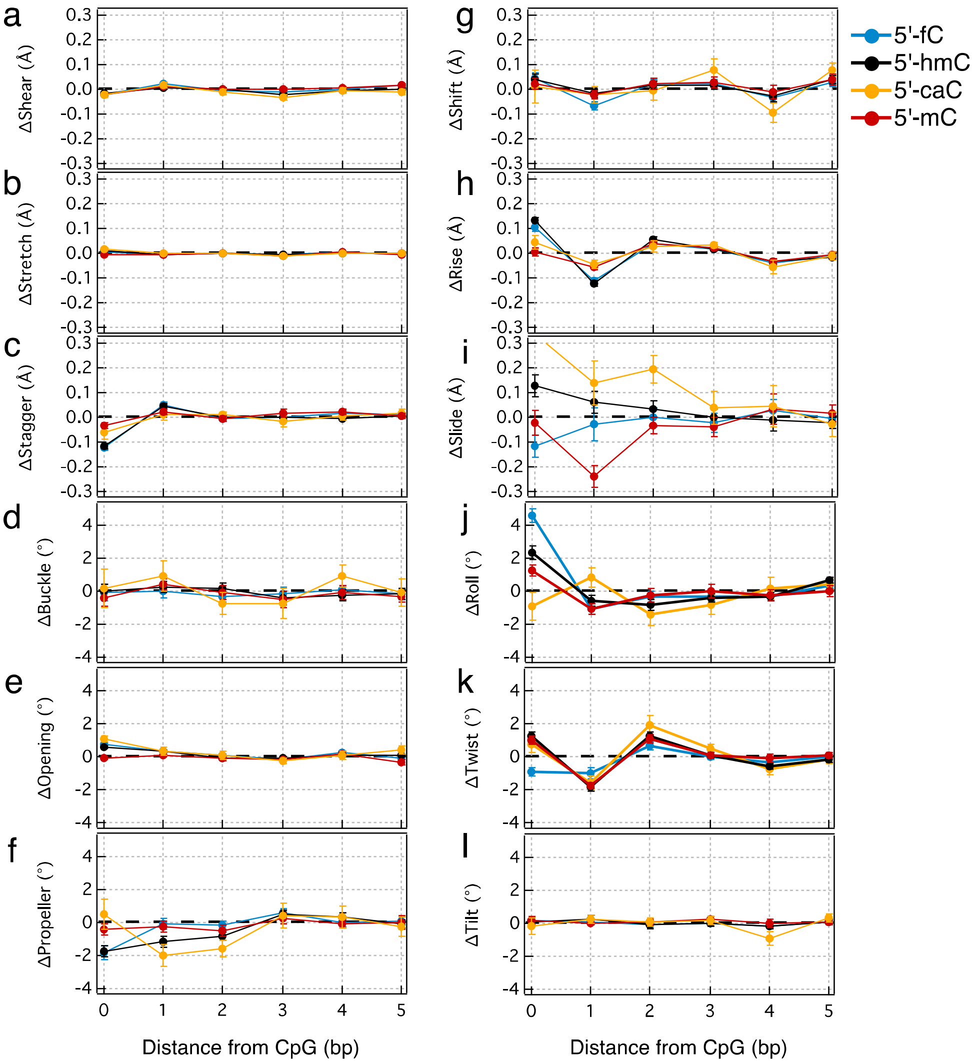

Supplementary Figure 2: Changes in the intra-base pair (**a-f**) and inter-base pair (**g-l**) parameters relative to the values of unmodified CpG sites as a function of the distance from the C·G base pair (**a-f**) or the center of the CpG base pair (**g-l**). Each data point represents an average over the four CpG steps, and over the corresponding MD trajectory block-averaged with a 20 ns interval. The error bars are the standard errors of mean.

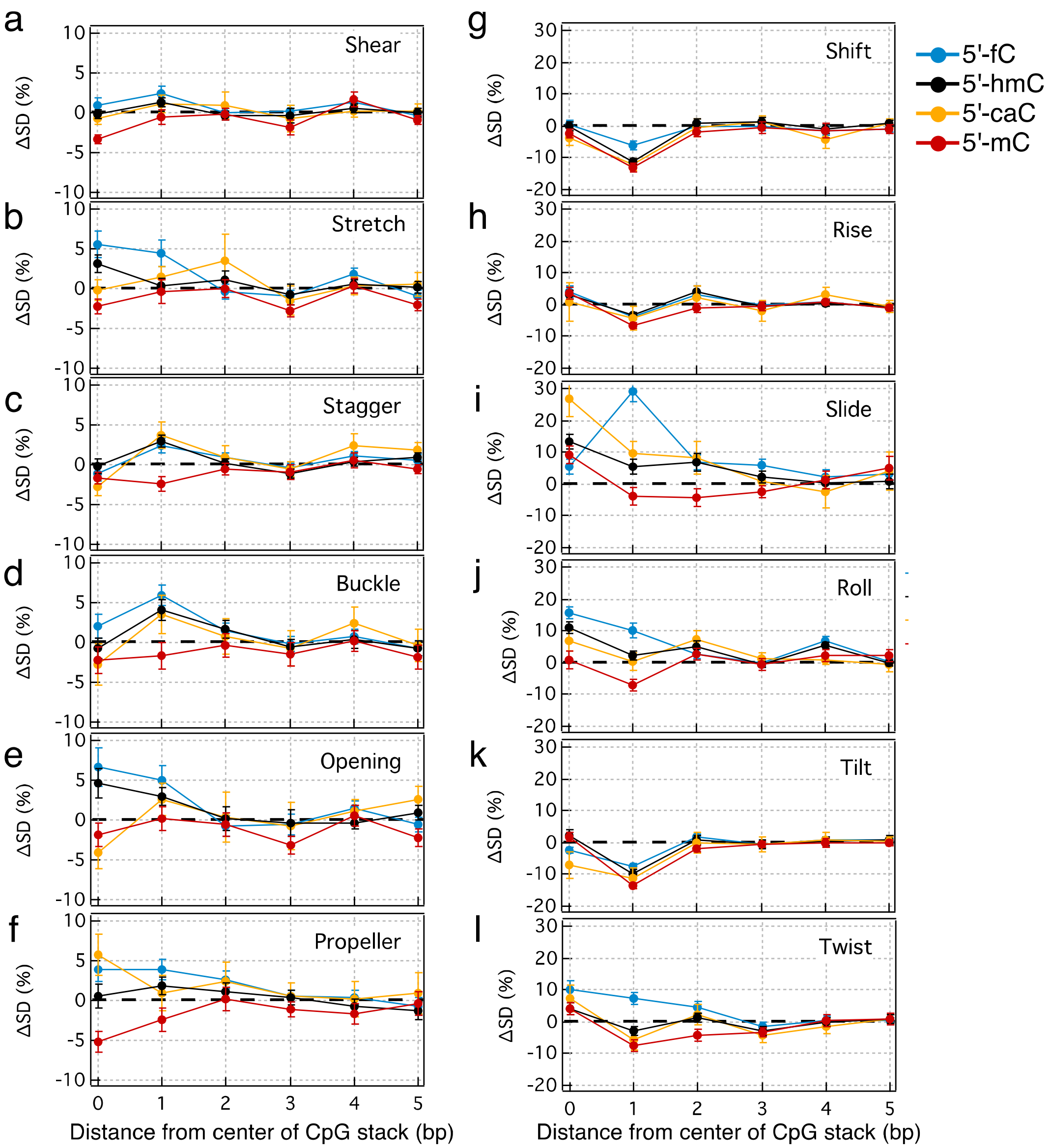

Supplementary Figure 3: Changes in the standard deviations of the intra-base pair (**a-f**) and inter-base pair (**g-l**) parameters relative to the values of unmodified CpG sites as a function of the distance from the C·G base pair (**a-f**) or the center of the CpG base pair (**g-l**). Data from four CpG sites were averaged. Each data point represents an average over the four CpG steps, and over the corresponding MD trajectory block-averaged with a 20 ns interval. The error bars are the standard errors of mean.

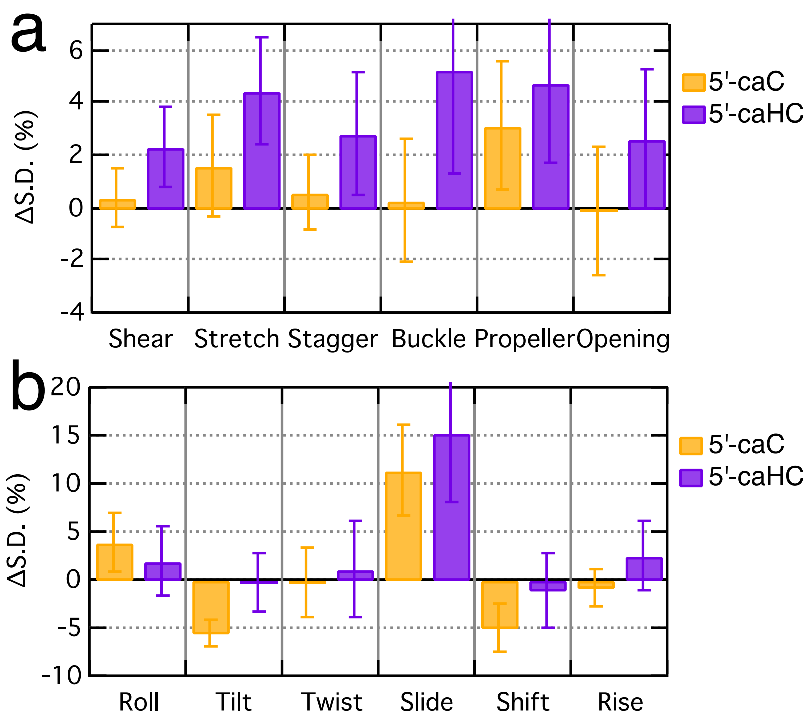

Supplementary Figure 4: The effects of protonated caC (caHC) modification of cytosine on of structural fluctuations of DNA. The average change in the standard deviation of the inter-basepair (**a**) and intra-basepair (**b**) structural parameters relative to the corresponding values of unmodified DNA. Data for the caC modifications (same as in the main text Figure 2) are shown for comparison. Each data point represents an average over three basepairs centered at a CpG step, over the four CpG steps, and over the corresponding MD trajectory block-averaged with a 20 ns interval. The error bars are the standard errors of mean. The simulations of the DNA systems containing the caHC modifications were performed using the same protocols as for the other systems.

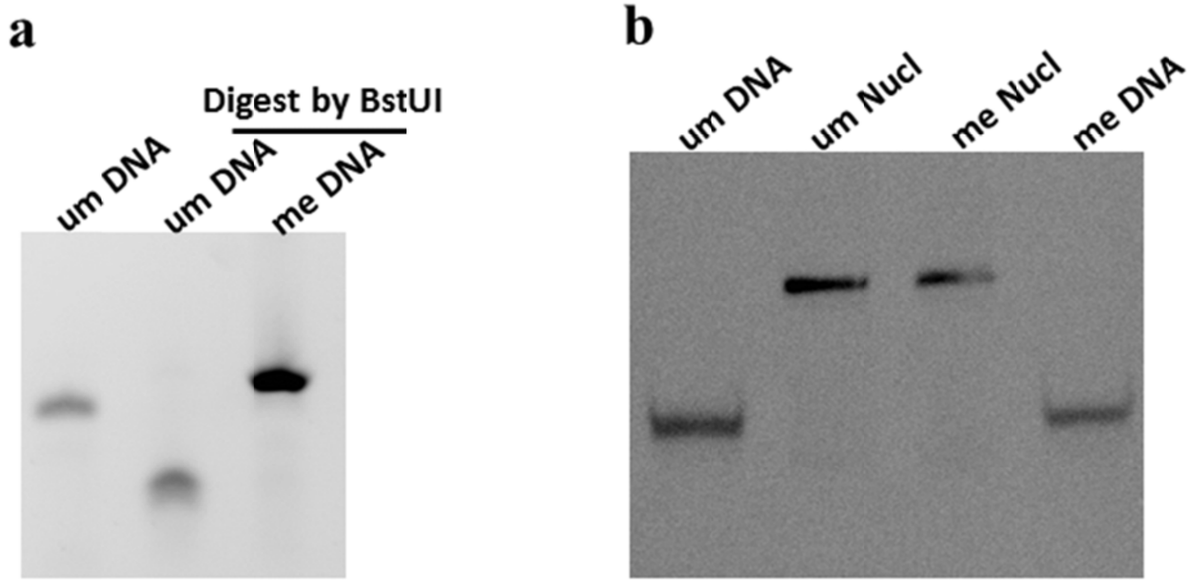

Supplementary Figure 5: Verification of methylation efficiency (**a**) and formation of nuclesosome on methylated DNA (**b**) by 5 % native PAGE gel.

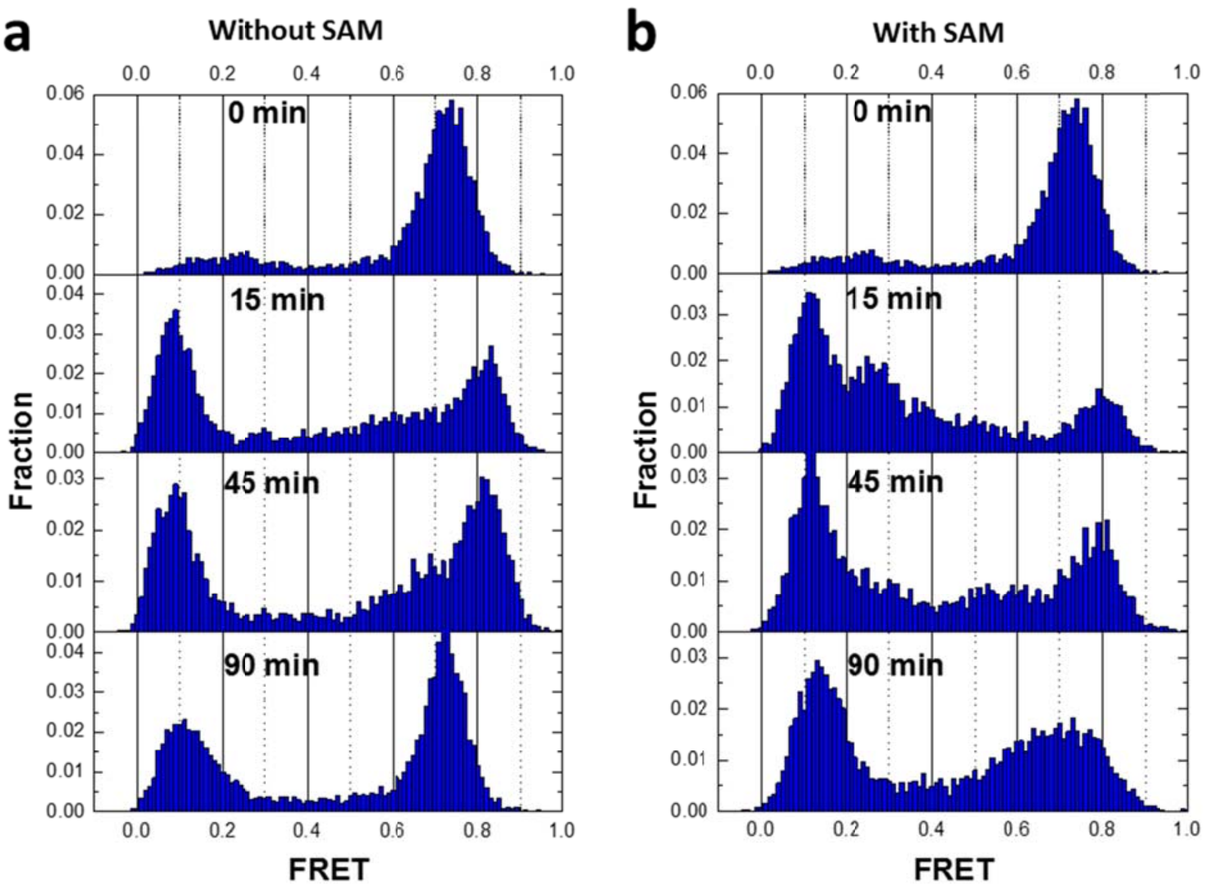

Supplementary Figure 6: FRET histogram of IJ-1 nucleosome during incubation with methyltransferase enzyme MsssI without (**a**) and with (**b**) SAM (S-adenosylmethionine). FRET peak shifts from 0.73 to 0.83 after MsssI is added (see 15 min time point) and returns to 0.73 after 90 min likely due to MsssI dissociation (a). With SAM present, FRET distributions are substantially different, likely due to the methylation reaction (b).

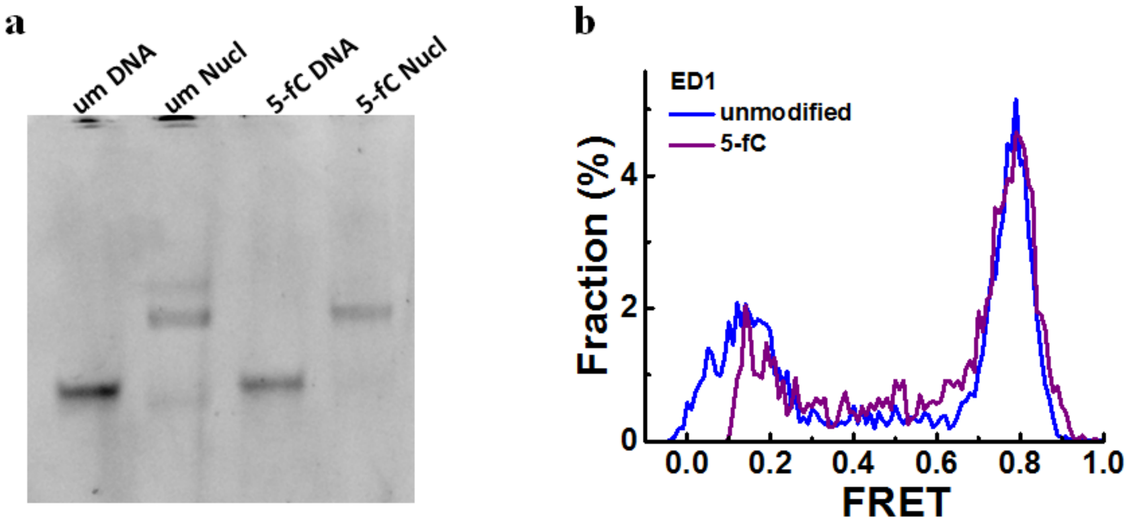

Supplementary Figure 7: Verification of the formation of nucleosomes on unmodified DNA and DNA containing two copies of 5-fC by 5% native PAGE gel (**a**) and FRET histogram (ED1 labeling scheme (**b**).
